# Computational deconvolution of genome wide expression data from Parkinson's and Huntington's disease brain tissues using population-specific expression analysis

**DOI:** 10.3389/fnins.2014.00441

**Published:** 2015-01-09

**Authors:** Alberto Capurro, Liviu-Gabriel Bodea, Patrick Schaefer, Ruth Luthi-Carter, Victoria M. Perreau

**Affiliations:** ^1^Department of Cell Physiology and Pharmacology, University of LeicesterLeicester, UK; ^2^Neural Regeneration Unit, Institute of Reconstructive Neurobiology, University of BonnBonn, Germany; ^3^Clem Jones Centre for Ageing Dementia Research, Queensland Brain Institute, The University of QueenslandSt Lucia, QLD, Australia; ^4^DFG-Center for Regenerative Therapies DresdenDresden, Germany; ^5^The Bioinformatics Core and The Synaptic Neurobiology Laboratory, The Florey Institute of Neuroscience and Mental HealthParkville, VIC, Australia

**Keywords:** computational deconvolution, Huntington's disease, Parkinson's disease, autophagy, microarray, transcriptomic analysis

## Abstract

The characterization of molecular changes in diseased tissues gives insight into pathophysiological mechanisms and is important for therapeutic development. Genome-wide gene expression analysis has proven valuable for identifying biological processes in neurodegenerative diseases using post mortem human brain tissue and numerous datasets are publically available. However, many studies utilize heterogeneous tissue samples consisting of multiple cell types, all of which contribute to global gene expression values, confounding biological interpretation of the data. In particular, changes in numbers of neuronal and glial cells occurring in neurodegeneration confound transcriptomic analyses, particularly in human brain tissues where sample availability and controls are limited. To identify cell specific gene expression changes in neurodegenerative disease, we have applied our recently published computational deconvolution method, population specific expression analysis (PSEA). PSEA estimates cell-type-specific expression values using reference expression measures, which in the case of brain tissue comprises mRNAs with cell-type-specific expression in neurons, astrocytes, oligodendrocytes and microglia. As an exercise in PSEA implementation and hypothesis development regarding neurodegenerative diseases, we applied PSEA to Parkinson's and Huntington's disease (PD, HD) datasets. Genes identified as differentially expressed in substantia nigra pars compacta neurons by PSEA were validated using external laser capture microdissection data. Network analysis and Annotation Clustering (DAVID) identified molecular processes implicated by differential gene expression in specific cell types. The results of these analyses provided new insights into the implementation of PSEA in brain tissues and additional refinement of molecular signatures in human HD and PD.

## Introduction

**Figure 1:**
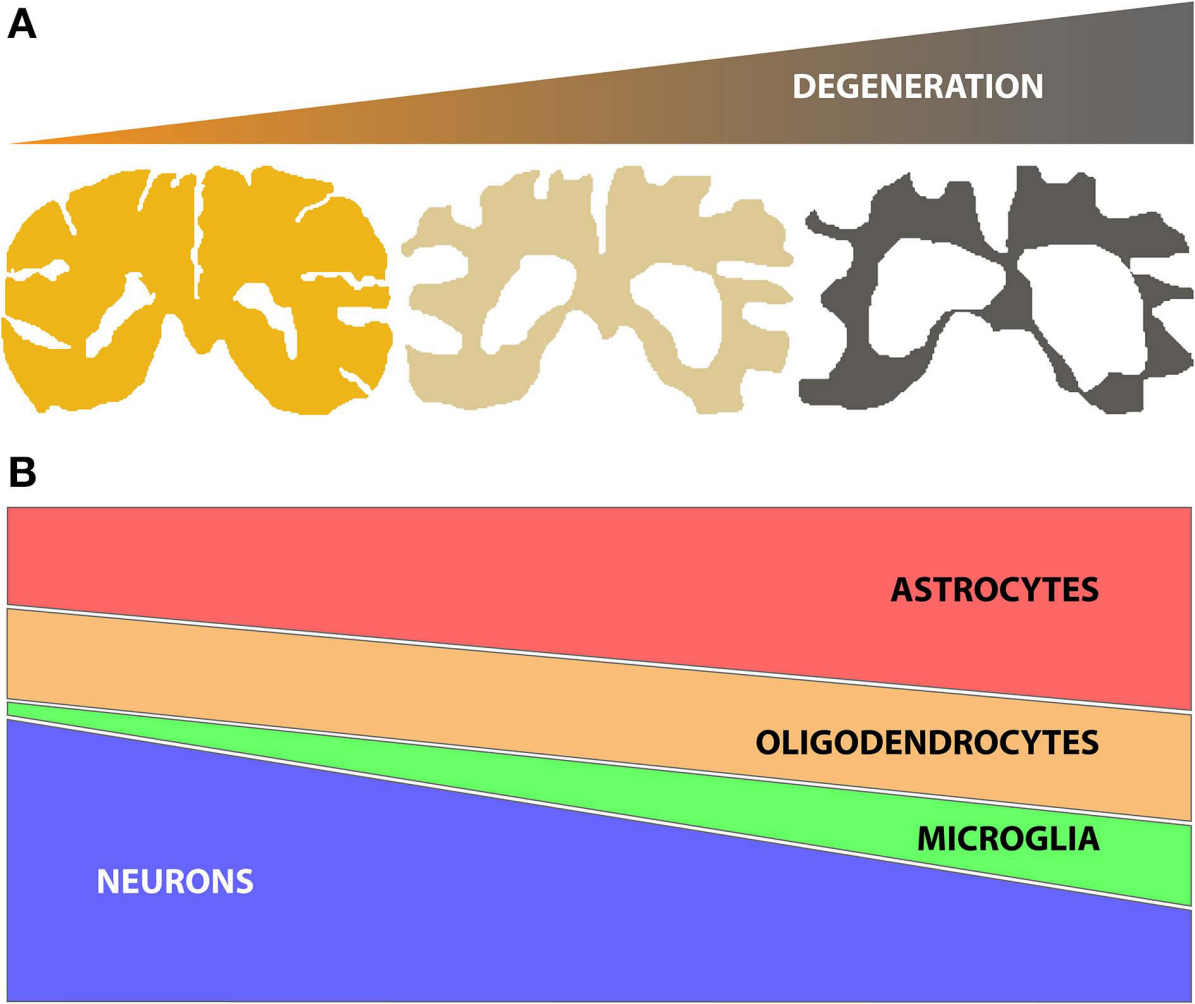
**(A)** Schematic representation of brain tissue atrophy concomitant with the progression of neurodegenerative disease; **(B)** Representation of the typical changes in the numbers of specific brain cell subtypes (neurons, oligodendrocytes, microglia and astrocytes) during disease, based on histopathological studies (Vonsattel et al., 2011). Of note, neuronal loss (atrophy and/or decrease in number) is accompanied by gliosis (increases in numbers of astrocytes, oligodendrocytes and/or microglia). The relative changes in specific glial subtypes vary by disease, however.

Identifying changes in gene or protein expression has the potential to focus attention on key molecular mechanisms underlying a given degenerative process (e.g., disease or aging effects on the brain). Genome wide expression studies using microarrays and next generation sequencing (NGS) technologies have been widely adopted to identify gene expression changes in human post mortem tissue for a number of neurodegenerative diseases. However, neurodegenerative diseases often lead to progressive changes in brain parenchyma composition, typically comprising a decline in the number of neuronal cells, together with an increase of glial cell number (astrocytes, oligodendrocytes and/or microglia) (Figure 1). These changes in the relative proportions of different cell populations can confound the ability to detect the molecular changes occurring in specific cell types. Therefore, when analyzing genome wide expression data from central nervous system (CNS) tissues it is important to use methods that can reliably account for changes in cell numbers to allow correct interpretations.

To overcome this problem, we have recently developed a method called Population-Specific Expression Analysis (PSEA) and shown its potential to successfully identify novel gene expression changes in human HD caudate (Kuhn et al., 2011). The method deconvolves brain expression heterogeneity by linear regression modeling to resolve cell-type-specific expression changes. We therefore reasoned that implementation of PSEA has the potential to create new, improved analyses of other brain datasets describing neurodegenerative processes.

Here we applied the PSEA method on publically available genome wide expression data generated from human Huntington's disease (HD) motor cortex (Hodges et al., 2006) as well as Parkinson's disease (PD) datasets derived from human prefrontal cortex, putamen and substantia nigra tissues (Zhang et al., 2005). Differentially expressed genes were analyzed for their inclusion in co-regulated gene networks, protein-protein interactions (PPIs) and memberships within identified functional networks to identify biological processes that may underlie disease-related effects. These analyses reinforced the robustness of the method and identified potential genes and pathways for further study.

## Materials and methods

### Microarray gene expression data

In the present study, we applied PSEA and standard differential expression analyses to HD brain mRNA expression data from frontal cortex (BA4) samples (16 control and 18 HD brains) (Hodges et al., 2006), and to PD brain expression data from prefrontal cortex (15 control and 14 PD), substantia nigra (18 control and 11 PD) and putamen (15 control and 20 PD) (Zhang et al., 2005). Microarray data sets were downloaded from Gene Expression Omnibus (Barrett et al., 2013) (GSE3790 for HD and GSE20295 for PD) (http://www.ncbi.nlm.nih.gov/geo/). These data were generated on Affymetrix Human microarrays U133A (HD, PD) and U133B (HD only). Probesets were annotated with current HUGO Gene Nomenclature Committee (HGNC) gene assignments using bioDBnet (Mudunuri et al., 2009) (http://biodbnet.abcc.ncifcrf.gov/db/db2db.php#biodb).

### PSEA analyses

For a comprehensive review on the implementation of PSEA, see (Kuhn et al., 2015) and the PSEA R package (www.bioconductor.org). The initial step in the application of PSEA to the datasets was to select appropriate marker gene probesets for each brain cell population (neurons, astrocytes, oligodendrocytes and microglia) (Kuhn et al., 2011, 2012). Marker genes were selected based upon prior evidence of cell-type-restricted expression in the literature and previous implementation of PSEA in HD (Kuhn et al., 2011). We verified that the signals corresponding to different probesets representing the same cell population were highly correlated (mean correlation >0.5), and conversely, that the signals of the marker gene probesets representing the other cell populations were poorly correlated (mean correlation <0.05). The probesets used as reference expression signals for each cell type and dataset were as follows. For HD BA4 cortex, neurons: 221805_at (*NEFL*), 221801_x_at (*NEFL*), 221916_at (*NEFL*), 201313_at (*ENO2*), 210040_at (*SLC12A5*), 205737_at (*KCNQ2*), 210432_s_at (*SCN3A*); astrocytes: 203540_at (*GFAP*), 210068_s_at (*AQP4*), 210906_x_at (*AQP4*), 201667_at (*GJA1*), oligodendrocytes: 211836_s_at (*MOG*), 214650_x_at (*MOG*), 216617_s_at (*MAG*), 207659_s_at (*MOBP*), 207323_s_at (*MBP*), 209072_at (*MBP*); and microglia: 204192_at (*CD37*), 215051_x_at (*AIF1*), 209901_x_at (*AIF1*), 213095_x_at (*AIF1*). For PD prefrontal cortex, neurons: 221805_at (*NEFL*), 221801_x_at (*NEFL*), 221916_at (*NEFL*), 201313_at (*ENO2*), 210040_at (*SLC12A5*), 205737_at (*KCNQ2*); astrocytes: 203540_at (*GFAP*), 210068_s_at (*AQP4*), 210906_x_at (*AQP4*), 201667_at (*GJA1*); oligodendrocytes: 211836_s_at (*MOG*), 214650_x_at (*MOG*), 216617_s_at (*MAG*), 207323_s_at (*MBP*), 209072_at (*MBP*); and microglia: 215051_x_at (*AIF1*), 209901_x_at (*AIF1*), 213095_x_at (*AIF1*). For PD substantia nigra and putamen, neurons: 221805_at (*NEFL*), 221801_x_at (*NEFL*), 221916_at (*NEFL*), 201313_at (*ENO2*), 210040_at (*SLC12A5*), 205737_at (*KCNQ2*); astrocytes: 203540_at (*GFAP*), 210068_s_at (*AQP4*), 210906_x_at (*AQP4*), 201667_at (*GJA1*); oligodendrocytes: 211836_s_at (*MOG*), 214650_x_at (*MOG*), 216617_s_at (*MAG*), 207323_s_at (*MBP*), 209072_at (*MBP*); microglia: 215051_x_at (*AIF1*), 209901_x_at (*AIF1*), 213095_x_at (*AIF1*).

To calculate the reference expression signals for each cell type we proceeded as follows. First, the expression values of the selected probesets were normalized to an average value of 100 to give them an equal weight, and those reporting the same marker gene expression were then averaged to obtain a marker gene expression measure for each gene. Then we averaged the marker gene expression measures for each cell population to obtain a reference expression signal for each cell population for each sample.

The next step was to fit candidate multiple regression models for the data for all probesets. Using the Akaike information criterion (AIC) (Akaike, 1974), we selected the best model for each probeset in terms of the regressors to be included from the set of reference signals corresponding to the 4 cell populations considered. In this way, we obtained models with 1–4 regressors that were then tested for quality of fit. The criteria used were the following: *F*-test (*p* < 0.05), adjusted R squared >0.6 and Shapiro test for a Gaussian distribution of the residuals (*p* > 0.01). In addition, models with large intercepts (absolute value larger than half of the mean expression value of the probeset) or negative coefficients were excluded. The probesets comprising the reference expression signals were also removed from the final tables.

In order to test for differential expression between control and disease samples we added one auxiliary regressor to each of the selected models. This regressor was formed by a vector having zeros in all positions except for the ones of the disease samples corresponding to the interrogated cell type. The auxiliary regressors were added one at a time to assess the specific expression of each cell type included in the model. For each cell type, the regressor coefficient represents the specific expression in the control group, while the coefficient of the auxiliary regressor estimates the differential expression and the specific expression in the disease is determined as the sum of both. The quality of the models including the auxiliary variable was then re-assessed as explained above. Finally, we constructed a table showing the multiple regression results of the probesets ordered by the *p*-values of the differential expression, including only cases with *p* < 0.05. The PSEA was implemented using a customized R script (Gentleman et al., 2004) using the functions lm and stepAIC.

An alternative approach to select the multiple regression models was applied in tissues where the simultaneous modeling of expression in the four cell types was hampered by poor correlations of the expression reported by the astrocyte and oligodendrocyte reference probesets (see below). In these cases, we restricted our analyses to probesets that reported expression exclusively in a single cell type, so as to avoid misassignment of differential expression. Single cell type expression was assigned by computing the correlation between each probeset and the reference signals and restricting further analyses to the cases where the correlation was larger than 0.8 for a single cell type (e.g., neurons) and less than 0.2 for the other three cells types (e.g., astrocyte, oligodendrocyte and microglia). We then calculated regressions with only one cell-type regressor (neurons in the above example) plus the corresponding auxiliary regressor (for differential expression between disease and control). The models obtained in this way were tested for quality of fit in the same manner explained above for the models selected with AIC, and tables to show the differential expression results were constructed. This approach proved useful in cases where the probes typically used to create the reference expression signals showed poor correlation between them (as was observed for astrocyte and oligodendrocyte signatures in the PD substantia nigra and putamen data sets) because it allowed us to nonetheless apply the PSEA to the cell types that have good quality reference expression signals (neurons and microglia in this example). The disadvantage of this approach is that we are unable to make interpretations regarding the differential expression of genes that are expressed in multiple cell types or in the cell types with poorly correlated probesets comprising the reference expression signals (here, astrocytes and oligodendrocytes). As a way to validate the approach, we compared the performance of both strategies in selecting models using PD prefrontal cortex, which has good reference signals and a large number of differentially expressed genes. This showed that 70% of the differentially expressed probes identified by the standard implementation of PSEA were also detected using the alternative approach.

### Standard gene expression analysis

In order to evaluate the potential for PSEA to refine gene expression measures, we compared PSEA results to standard differential expression analyses performed with packages from Bioconductor (affy (Gautier et al., 2004) and limma (Smyth, 2005)) (www.bioconductor.org). HD cortex analysis with limma identified 1925 differentially expressed (DE) probesets passing a fdr cutoff of *p* < 0.05, consistent with our previous analysis of these data (Hodges et al., 2006). For PD cortex, limma analysis identified 304 DE probesets passing a fdr cutoff of *p* < 0.05, whereas 91 probesets showed differential expression in the substantia nigra and only 1 probeset in the putamen by the same significance threshold criterion.

### Validation of PSEA Assignment using cell specific expression data

Two independent, publically available genome-wide expression data sets examining mouse CNS cell type gene expression were used to evaluate PSEA-based expression predictions. These two datasets examined relative expression in three of the four CNS cell types examined in our the PSEA analyses (neurons, oligodendrocytes, astrocytes). One of the datasets employed subcultures of primary mouse brain cells to derive cell-type-specific expression profiles (Cahoy et al., 2008), of which we used profiles of postnatal day 16 neurons, postnatal day 17 astrocytes, and myelinating oligodendrocytes. The second data set employed translating ribosome affinity purification (TRAP) to mouse brain tissue to generate cell-type-specific expression profiles of different CNS cell types (Doyle et al., 2008), of which we used Ntsr1-positive cortical neurons, Aldh1L1-positive cortical astrocytes, and Cmtm5-positive cortical oligodendrocytes. Both datasets were generated using the Affymetrix Murine 430 plus 2.0 microarray platform. To compare our PSEA-based expression predictions from human tissues to the mouse data, murine probesets were annotated with current corresponding HGNC gene assignments using dbOrtho tool in bioDBnet (Mudunuri et al., 2009) and the list of probesets for which PSEA achieved suitable expression models (Tables S1, S2) were reduced to unique HGNC ids. Then for each assignment of expression in a particular cell type by PSEA, we tallied whether expression was detected in that cell type in the mouse datasets, using a threshold of >100 arbitrary normalized expression units.

### Validation of PSEA differentially expressed genes in PD substantia nigra

PSEA results of DE in PD substantia nigra neurons were validated using additional microarray expression data. These data were published in three independent studies that used laser capture microdissection (LMD) to selectively sample substantia nigra neurons; the tissue samples in these studies were comparable in terms of patient cohort age and disease state to the samples used in the PSEA analysis. For each of the 8 genes identified as differentially expressed in substantia nigra by PSEA (*p* < 0.05) we determined if the gene was identified as significantly changed in the same direction in any of the three LMD data sets. For two of the LMD studies (Simunovic et al., 2010; Elstner et al., 2011) fold change and *p*-values from microarray gene expression analyses available as supplementary data with the original manuscript were used for comparison. DE results were not published for the third LMD data set (Middelton-1 dataset included in Zheng et al., 2010); therefore the raw microarray data was downloaded from Gene Expression Omnibus (accession number GSE20141) and we performed differential expression analyses by standard limma analysis to obtain the necessary statistics (Smyth, 2005). LMD *p*-values and fold changes for all three studies are tabulated for comparison against PSEA-determined values.

### Functional annotation clustering

The David 2.0 Bioinformatics database was used to identify functionally related groups of DE genes in individual cell types (http://david.abcc.ncifcrf.gov/) (Huang Da et al., 2009; Sprooten et al., 2014). Panther and Reactome pathways were added to the default selected functional annotation and the functional annotation clustering tool was applied to the lists of probe sets. The classification stringency setting used was medium with default setting for function grouping except for enrichment thresholds for EASE which were reduced to 0.05 to reduce inclusion of non significant terms into the clusters. Annotation Clusters of significantly over represented groups with terms having a FDR <50% were accepted for further consideration.

### Network analysis

Cytoscape was used to examine coregulation, cell type specific expression and PPI networks for DE genes. A HGNC database was downloaded in July 2014 (Gray et al., 2013) (http://www.genenames.org/) and imported into Cytoscape 2.8.3 (http://www.cytoscape.org/) (Smoot et al., 2011). Using HGNC gene symbols as the key ID, the HGNC network was then merged with a network of coregulated gene pairs computed from seven normal human cortex data sets (Mistry et al., 2013). Human PPIs for DE genes (proteins) within the selected network were retrieved from Biogrid on August 17th 2014 (http://thebiogrid.org/) (Stark et al., 2006). Publically available genome wide gene expression data of mouse CNS cell types was obtained as supplementary data (Doyle et al., 2008) and mapped onto the HGNC network after retrieving the current corresponding HGNC gene assignments using dbOrtho tool in bioDBnet (Mudunuri et al., 2009). Cytoscape App Multicolored nodes (Warsow et al., 2010) was employed to visualize cell specific expression data in specific molecular networks.

## Results and discussion

The present analyses comprise a study in the implementation of PSEA within the International Neuroinformatics Coordinating Facility (INCF) Short Course on Neuroinformatics, Neurogenomics and Brain Disease held in 2013 (https://sites.google.com/site/neuroinformaticsjamboree), with a view toward assessing candidate mechanisms of human neurodegenerative diseases. The datasets used in the analyses presented here are publically available and derived from postmortem samples of human HD (Hodges et al., 2006) and PD brains (Zhang et al., 2005) (see Materials and Methods). These two conditions represent two functionally distinct disease-related perturbations to the motor output circuit involving the cerebral cortex and basal ganglia (Albin et al., 1989).

HD is an autosomal dominantly inherited neurodegenerative disorder caused by a CAG repeat expansion in the Huntingtin (HD, ITI5) gene (The Huntingtons Disease Collaborative Research Group, 1993). The CAG repeat expansion encodes a polyglutamine stretch within the N-terminal domain of the huntingtin protein (Htt) that imparts neurotoxicity, mostly through gain-of-function mechanisms, including Htt protein aggregation, as has been recapitulated in animal studies (Bates et al., 2002). A recent report using the PSEA method corroborated previous mouse data by detecting myelin-related gene expression changes in HD oligodendrocytes, supporting the possibility that deficient myelin synthesis and/or composition changes may by drivers of disease in HD (Kuhn et al., 2011).

PD is a neurodegenerative disease that can have either a sporadic or a familial etiology (Block et al., 2007). PD is typified by specific loss of dopaminergic neurons of the substantia nigra pars compacta that project to the neostriatum (caudate nucleus and putamen) (Dickson, 2012). This degeneration leads to PD-related symptoms including bradykinesia, tremor, rigidity and postural instability (Dauer and Przedborski, 2003). At the cellular level, PD is characterized by α-synuclein protein aggregates in neuronal cells (also known as Lewy bodies). Genes associated with familial PD include the gene encoding α-synuclein (*SNCA*) and genes involved in protein turnover and mitochondrial homeostasis, such as *PARK2*, which encodes Parkin (Kumar et al., 2012).

### Implementation of PSEA to identify differential expression within specific cell subpopulations in HD and PD brains

In the present study, we applied PSEA analyses to expression data derived from motor cortex (BA4) samples from 16 control and 18 HD brains, and to prefrontal cortex samples from 15 control and 14 PD brains. For these cortical samples, the PSEA expression models were selected with the approach described in (Kuhn et al., 2011; see Materials and Methods) to assign expression and analyze differential expression using a model representing four cell compartments (neurons, astrocytes, oligodendrocytes and microglia). The regressors used for each of these models are listed in Supplementary Tables S1, S2. For HD motor cortex, PSEA provided evidence of 14 genes differentially expressed in neurons, 6 in astrocytes, and 8 in oligodendrocytes (0 in microglia) (Table S3). For PD prefrontal cortex, PSEA identified differential expression of 122 genes in neurons, 5 in astrocytes, 11 in oligodendrocytes, and 11 in microglia (Table S4). There was very little overlap between the genes identified as DE in HD and PD cortex but, interestingly, both diseases shared down regulation of doublecortin-like kinase 1 (*DCLK1*), a multi-functional, neuronally expressed gene known to be involved in neuronal migration, retrograde transport, neuronal apoptosis and neurogenesis (Dijkmans et al., 2010).

When the same PSEA procedures were applied to the PD substantia nigra (18 control and 11 PD) and putamen (15 control and 20 PD) datasets, we observed that the expression signals for the probesets that we had previously used to represent astrocytes and oligodendrocytes did not exhibit good correlation. Therefore, an alternative approach was adopted to select the multiple regression models that could be fit to a single cell type (neurons or microglia only), to avoid relying on reference signals with poor correlations (see Materials and Methods). These analyses provided evidence for the differential expression of 8 genes in neurons in the substantia nigra (Table S5) and 11 genes in neurons in the putamen (Table S6). Interestingly, however, *DCLK1* was again one of the genes identified as differentially expressed in neurons in the substantia nigra, and was thus detected as differentially expressed in three of four neuronal datasets.

### External validation of assignment of cellular expression

We used publically available genome wide expression datasets to validate PSEA-based expression assignments. Comprehensive cell type expression data generated from human cells was not available so expression data from mouse models were used (see Materials and Methods Section “Validation of PSEA Assignment using Cell Specific Expression Data”). We were able to corroborate PSEA-assigned expressions for a very large fraction of genes for which a suitable four-cell-type expression model could be constructed (Table 1).

**Table 1:** **Supporting evidence for gene expression in specific cell types from publically available datasets**.

**Disease**	**Cell type**	**Number of regressor probe sets**	**Number of unique HGNC IDS**	**Mouse HGNC homologs with expression > 100**	
				**Primary culture expression (Cahoy et al., 2008)**	**TRAP expression (Doyle et al., 2008)**
Huntington's disease cortex	Neuron	282	267	81% (210/259)	75% (193/259)
	Astrocyte	60	54	84% (43/51)	73% (37/51)
	Oligodendrocyte	194	179	80% (139/174)	66% (115/174)
Parkinson's disease cortex	Neuron	261	254	78% (188/239)	70% (167/239)
	Astrocyte	89	88	75% (62/83)	64% (64/83)
	Oligodendrocyte	68	65	67% (40/60)	53% (32/60)

*PSEA assignments of gene expression in neurons, astrocytes and oligodendrocytes were verified using two independent, publically available mouse expression datasets (Cahoy et al., 2008; Doyle et al., 2008). For procedures, see Materials and Methods, Section Validation of PSEA assignment using cell specific expression data*.

### Comparison of PSEA and standard differential expression analyses

**Figure 2:**
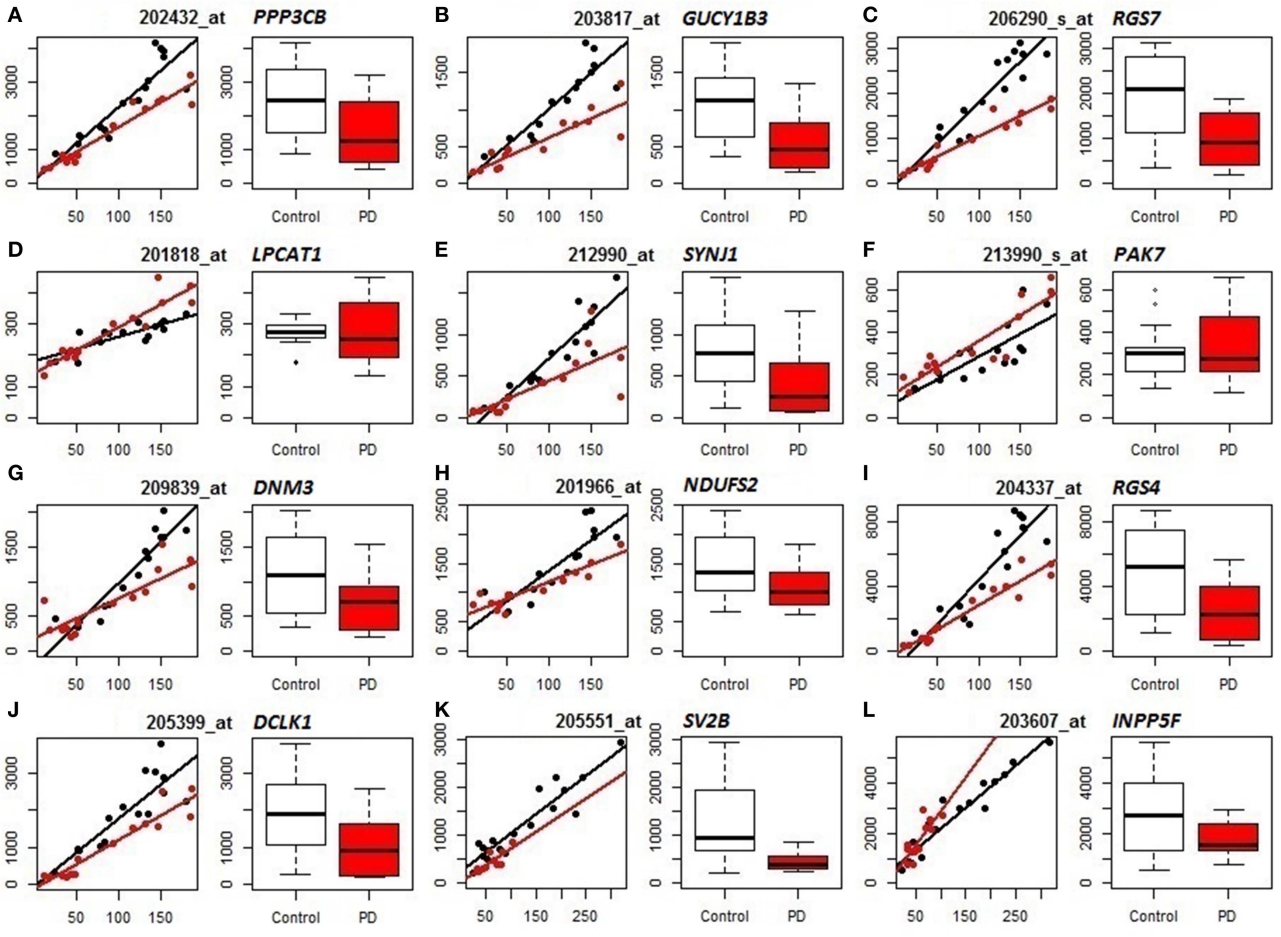
**Comparison of PSEA-derived expression changes (left panels, regression plots) and standard gene expression measures (right panels, bar graphs) in PD (Control samples shown in black, PD samples in red)**. **(A–J)** Neuronal expression in PD prefrontal cortex. **(K,L)** Neuronal expression in PD substantia nigra. For each probeset (mRNA/gene) we present 2 panels showing its neuron-assigned expression plotted against the neuron reference expression signal for each sample (where the differential expression can be visualized by the difference in slopes, left panels) and box plots directly comparing the expression values (right panels). PSEA statistics for each gene can be found in Table S4. Limma statistics for each gene are as follows. **A**: log fold change = −0.862, *p* = 0.011, FDR *p*-value 0.145, **B**: log fold change = −1.1, *p* = 0.002, FDR *p*-value = 0.07, **C**: log fold change = −1.184, *p* = 0.002, FDR *p*-value = 0.078, **D**: log fold change = −0.034, *p* = 0.822, FDR *p*-value = 0.914, **E**: log fold change = −1.449, *p* = 0.005, FDR *p*-value = 0.103, **F**: log fold change = 0.065, *p* = 0.785, FDR *p*-value = 0.895, **G**: log fold change = −0.71, *p* = 0.043, FDR *p*-value = 0.245, **H**: log fold change = −0.388, *p* = 0.054, FDR *p*-value = 0.268, **I**: log fold change = −1.249, *p* = 0.011, FDR *p*-value = 0.144, **J**: log fold change = −1.272, *p* = 0.008, FDR *p*-value = 0.125, **K**: log fold change = −1.325, *p* = 0.0002, FDR *p*-value = 0.049, **L**: log fold change = −0.409, *p* = 0.231, FDR *p*-value = 0.646.

We subsequently compared the outputs of population-specific and standard expression analyses (limma) to assess the potential improvement of cellular resolution of DE using PSEA. (For reference, results of the limma analyses of the datasets are included as Table S7.) Example comparisons from the analyses of PD frontal cortex and substantia nigra are illustrated in Figure 2. In the left panels for each gene (mRNA) their neuron-assigned expression values are plotted against the neuron reference expression signals to visualize disease-related expression differences in the slopes of the linear regressions fitted to the individual sample datapoints (black for control, red for PD). The accompanying box plots show the uncorrected expression values (from limma). These plots indicate examples that distinguish true differential expression from the reduction in the overall numbers of neuronal cells [*SV2B* (Figure 2K)], cases where PSEA detects differential expression when standard analyses were equivocal [*PPP3CB* (Figure 2A), *GUCY1B3* (Figure 2B), *RGS7* (Figure 2C), *SYNJ1* (Figure 2E), *DNM3* (Figure 2G), *NDUFS2* (Figure 2H), *RGS4* (Figure 2I), *DCLK1* (Figure 2 **J**)] and examples where limma would mis-assign the trend for differential expression as decreased rather than increased in diseased vs. control neurons [*LPCAT1* (Figure 2D), *PAK7* (Figure 2F), and *INPP5F* (Figure 2L)].

### Comparison of DE genes in substantia nigra with laser capture microdissection data

Due to the strong interest in the effect of PD on neuronal gene expression in the substantia nigra pars compacta, a number of independent data sets have been published using LMD to obtain neuron-specific microarray expression profiles from postmortem human tissue. Although LMD cannot entirely replicate the PSEA analysis because it is limited to sampling RNA profiles in the cell body, whereas neuronal RNAs can be transported from the soma for local translation in both dendrites and axons, it is nonetheless the most appropriate data available for comparison. Despite this limitation, 6 out of 8 genes detected as differentially expressed in substantia nigra neurons by PSEA (Table S5) were validated as DE in the same direction in at least 1 independent LMD PD study. Furthermore, 4 out of 8 DE genes were validated in 2 independent studies (Table 2). These data further support the accuracy of prediction of cellularly resolved DE by PSEA.

**Table 2:** **Validation of PSEA determined DE in neurons in substantia nigra in PD by comparision with three independent LMD studies**.

**Gene symbol**	**PSEA analysis this study**		**Simunovic et al., 2010**		**Elstner et al., 2011**		**Zheng et al., 2010**	
	***p*-value**	**Log fold change**	***p*-value**	**Log fold change**	***p*-value**	**Log fold change**	***p*-value**	**Log fold change**
CHN1	6.10E-03	−1.61	< 0.01	−2.63			3.79E-02	−1.61
NSF	1.10E-02	−0.44	< 0.01	−3.23	7.74E-03	−1.15		
SV2B	1.44E-02	−0.87	< 0.01	−2.50			4.79E-02	−1.20
GABARAPL1	2.59E-02	−0.73	< 0.01	−1.31	1.10E-02	−1.31		
DCLK1	3.29E-02	−0.90					6.95E-03	−1.48
ATP6V1A	3.58E-02	−0.71	< 0.01	−3.33				

*Eight genes were identified as DE by PSEA and those that were also significantly DE in at least one independent study are included in the table. Fold changes and p-values obtained were from the published manuscript where possible (Simunovic et al., 2010; Elstner et al., 2011) or generated from re analysis of raw data (Zheng et al., 2010)*.

The genes identified by PSEA as decreased in expression in PD substantia nigra, and validated by independent LMD studies, namely, *chimerin 1* (*CHN1*) *N-ethylmaleimide-sensitive factor* (*NSF*), *Synaptic Vesicle Glycoprotein 2B* (*SV2B*), GABA(A) receptor-associated protein like 1 (*GABARAPL1*), the *proton-transporting lysosomal 70kDa protein ATPase subunit V1 subunit A* (*ATP6V1A*), and *DCLK1* comprise potential candidates for further investigation. The synaptic vesicle-associated protein encoded by *NSF* has been recently highlighted in another bioinformatics screen aimed at identifying novel therapeutic targets for PD that included a meta analysis of transcriptomic data (Karic et al., 2011). In addition, a recent GWAS study identified *NSF* as novel candidate PD susceptibility gene in an Ashkenazi Jewish population (Liu et al., 2011). *SV2B* was also decreased in HD cortex neurons and *DCLK1* was decreased in both HD and PD cortex neurons, suggesting that these genes may be involved in common pathways impacting neuronal degeneration in HD and PD. *GABARAPL1* is also decreased in PD putamen neurons and is a key gene in the regulation of autophagy (see below).

### Interesting genes and pathways represented in the PSEA results

The largest numbers of differentially expressed genes identified by our PSEA analyses in both HD and PD brains were in neuronal cells, although differences were also found in other brain cell types (astrocytes, oligodendrocytes and microglia). Annotation cluster analyses of the PSEA results using DAVID (Huang Da et al., 2009) and network analysis, employing both coregulation and PPI data, identified a number of genes and biological processes that have been implicated in neurodegenerative diseases.

#### Functional annotation cluster analyses in HD brain highlight endosome and plasma membrane signaling pathways

Htt is known to have functions in protein trafficking, vesicle transport, and postsynaptic signaling that may be altered by the HD-causing mutation (Gil and Rego, 2008). DAVID analyses of the genes detected as DE in HD neurons identified enrichment in genes related to vesicular transport and protein localization (Table 3, Annotation cluster 2). These included the endosomal Ras-related protein Rab-9B GTPase (*RAB9B*), syntaxin 1B (*STX1B*), the secretory carrier membrane protein 5 (*SCAMP5*), and the coiled-coil domain containing protein 91 (*CCDC91*). Interestingly, we have previously observed DE of *RAB9B* in a mouse model of HD (Tang et al., 2011). *STX1B* is essential for the regulation of spontaneous and evoked synaptic vesicle exocytosis (Mishima et al., 2014) and has been associated with PD in GWAS studies (Pihlstrom et al., 2013). *SCAMP5* is involved in vesicle endocytosis (Zhao et al., 2014) and has been associated with autism (Castermans et al., 2010). *CCDC91* is thought to be involved in white matter development and maintenance (Sprooten et al., 2014).

**Table 3:**
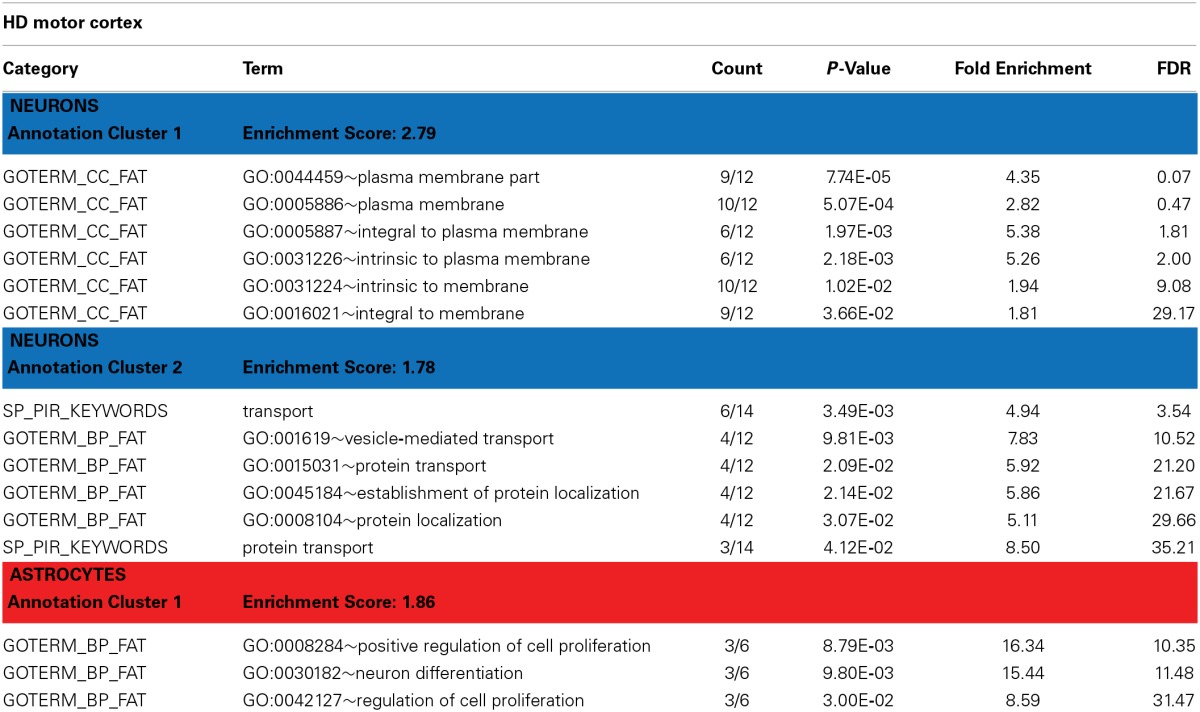
**David functional annotation clustering with differentially expressed genes in the motor cortex of HD brains (revealed by PSEA)**.

Annotation cluster 1 of neuronally DE genes in HD cortex (Table 3) is characterized by genes with plasma membrane-associated functions. This group includes G protein-coupled receptor 176 (GPR176), ADAM metallopeptidase domain 23 (*ADAM23*), synaptic vesicle glycoprotein 2B (*SV2B*), and *DCLK1*, but also *RAB9B, STX1B* and *SCAMP5*. Progressive abnormalities in SV2 expression in skeletal muscle and neuromuscular junctions have been previously reported in a mouse model of HD (Ribchester et al., 2004). *DCLK1* is involved in cortical development (Sossey-Alaoui and Srivastava, 1999) and has been associated with schizophrenia and attention deficit/hyperactivity disorders (Havik et al., 2012). ADAM23 is a cell-cell adhesion protein highly expressed in the nervous system (Goldsmith et al., 2004) and suspected to regulate neuronal differentiation (Wang et al., 2012).

#### Functional annotation cluster analyses in PD brain find mitochondrion-associated molecules

Within the PD datasets, genes encompassing a variety of functions previously associated with PD were identified as DE, including pathways common to a number of different neurodegenerative diseases, such as HD and Alzheimer's disease (AD) (Table S8, Annotation cluster 4).

Functional annotation clustering analysis of genes DE in PD cortical neurons exhibited the highest complexity of enriched elements. Among these was an abundance of purine nucleotide binding-related genes (Annotation clusters 1, and 3). The cortical neuron purine binding nucleotide cluster comprises 26 DE genes, including several tubulin genes (*TUBB2A, TUBG1, TUBA1B*). Annotation clusters 2 and 5 include many terms associated with microtubules and tubulin, reflecting that abnormalities in microtubule dynamics have also been previously implicated in PD (reviewed in Feng, 2006).

Several lines of evidence have jointly supported causal links between changes in mitochondrial energetics and function and neuron-specific degeneration in PD (Jin et al., 2006; Narendra et al., 2008; Schapira, 2008; Van Laar and Berman, 2009). Consistent with this idea, oxidative phosphorylation, mitochondrial biological processes and mitochondrial localization were evident in the PD cortical neuron DE (Table S8, Annotation cluster 4), being represented by the mitochondrial inner membrane protein (*IMMT*), NADH dehydrogenase (ubiquinone) 1 subcomplex unknown-2 (*NDUFC2*) or cytochrome c oxidase subunits 4 isoform 1 and 5a (*COX4I1* and *COX5A*). Changes in *COX* gene transcription has been purported to reflect cellular levels of oxidative stress (Roemgens et al., 2011) and affect cytochrome C oxidase activity (Castello et al., 2008). Interestingly, *COX4I1* and *COX5A* were also found DE in the microglial PD PSEA data (Table S4). Microglial cells are specialized phagocytes that react to neuronal injury or damage (Glass et al., 2010) and can release neurotoxic reactive oxygen species under certain conditions (Block et al., 2007). Together with the fact that dopaminergic neurons are more sensitive to oxidative stress compared with other types of neurons (Block et al., 2007), changes in cytochrome activity in neurons or microglia might have a proximal effect on the development of PD (Chaturvedi and Beal, 2013).

Kinase genes, including selected mitogen-activated protein kinase-related genes (*MAP2K1, MAP2K4, MAPK10*), were well-represented within neurons in the PD cortex (Table S8 Annotation cluster 3). During neuronal injury various MAPKs can be activated in relation with effects on cellular respiration, transport, release of reactive oxygen species, mitophagy and apoptosis (Dagda et al., 2009). DE kinases also included the calcium/calmodulin-dependent protein kinase II beta (*CAMK2B*), known to important for synaptic plasticity and memory (Shonesy et al., 2014) and a brain-enriched p21-activated kinase (*PAK7*), as well as *DCLK1*.

#### Network analyses based on coregulation and protein-protein interaction highlight autophagy-related DE genes in PD neurons

Analyses using publically available data of coregulation and PPIs were undertaken to provide a complementary way to identify functional groups of genes within lists of identified DE gene lists. Coregulation of genes was used to initially construct DE gene networks, to which we added shared DE gene (protein) interacting proteins to potentially identify common targets or regulators of DE genes (proteins). Data from CNS cell-type-specific expression data was also utilized. Together these approaches increased insight into the nature of a given network's function.

**Figure 3:**
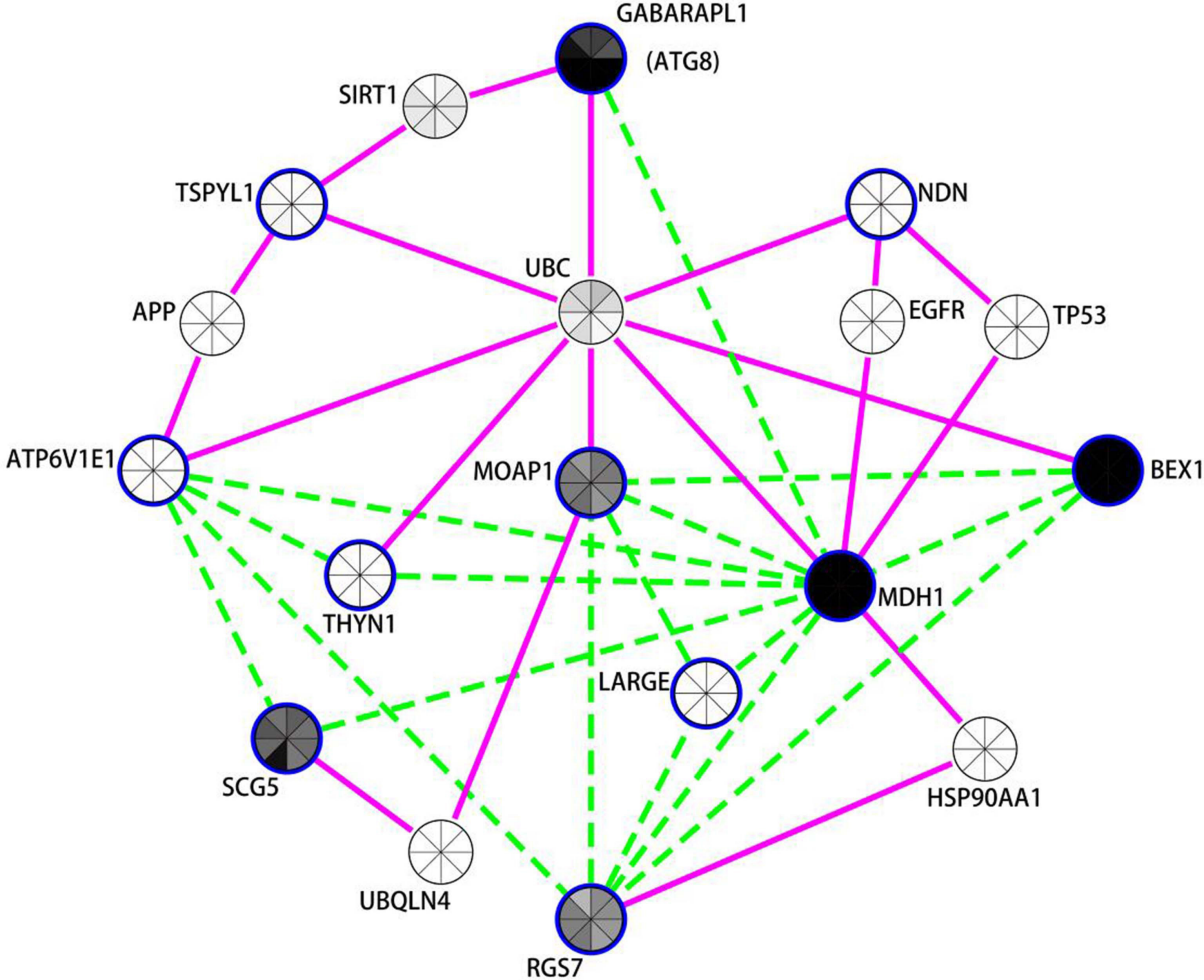
**Network analysis of differentially expressed neuronal genes in the putamen in PD indicates autophagy modulation**. Expression coregulation data, PPIs and gene expression levels in mouse neuronal cells strongly implicates the mammalian neuronal specific ATG8 homolog GAPARAPL1 and other vesicle associated genes in the regulation of autophagy in the putamen in PD. Blue borders indicate nodes for genes/proteins that were DE in PD putamen neurons. With the exception of *TSPYL1*, all were identified as decreased in PD compared to controls. Nodes without a blue border were introduced into the network due to evidence of common human PPIs with at least two DE genes. Gene expression coregulation within human frontal cortex (Mistry et al., 2013) is indicated by dashed green edges and PPIs are indicated by solid magenta edges. The centers of the nodes are also shaded by normalized translational ribosomal affinity profiling expression levels (TRAP) from eight different murine neuronal cell populations (Doyle et al., 2008). The neuronal populations are (clockwise from top); cortical Cck neurons, cortical Cort interneurons, striatal Drd1 medium spiny neurons, striatal Drd2 medium spiny neurons, cortical Etv1 corticostriatal neurons, corticospinal Glt25d2 neurons, cortical Ntsr1corticothalamic neurons, and cortical Pnoc interneurons. Gray indicates low expression and black high expression.

One particularly well-populated gene network, of DE genes in PD putamen neurons, is illustrated in Figure 3. This network shows strong representation of autophagy-related processes. Autophagy is a highly evolutionarily conserved process carried out by the endosomal-lysosomal system to regulate protein and organelle turnover via targeted lysosomal degradation. There is increasing evidence that abnormalities in autophagy may contribute to neurodegeneration in HD, PD and AD (Lynch-Day et al., 2012). In particular, many proteins related to PD have specific roles in the regulation of autophagy and/or mitophagy (the clearance of mitochondrial components by autophagy) (reviewed in De Vries and Przedborski, 2013). There is clear evidence that reduced autophagy can lead to PD-linked phenomena including accumulation of α-synuclein, mitochondrial dysfunction and neuronal death (reviewed in Zhang et al., 2014).

The autophagy-related network illustrated in Figure 3 prominently features *GABARAPL1*, an autophagy gene 8 (*ATG8*) homolog, which was decreased in both the substantia nigra and putamen in PD brains. *GABARAPL1* is the most highly expressed *ATG8* homolog in the nervous system and it encodes a key autophagy protein that associates with autophagic vesicles (Chakrama et al., 2010). Its expression is limited to neuronal cells, and it is particularly highly expressed in the substantia nigra pars compacta, the region most affected by Parkinson's disease (Le Grand et al., 2013). In the neuronal putamen DE gene network, *GABARAPL1* expression was coregulated with another highly expressed neuronal gene, Malate Dehydrogenase 1, NAD (*MDH1*). *MDH1* is important in transporting NADH equivalents across the mitochondrial membrane and also interacts with the ubiquitin ligase PARKIN, encoded by *PARK2* (Cookson, 2012; Scarffe et al., 2014). PARKIN is known to work together with PINK1 to promote mitophagy (Scarffe et al., 2014). Another mitochondrial gene, brain expressed, X-linked 1 (*BEX1*) (Xiao et al., 2014) interacts with the p75 neurotrophin receptor (Vilar et al., 2006) and is upregulated in axonal injury (Khazaei et al., 2010). Other genes in this network also play roles in synaptic vesicle exocyotosis [Regulator Of G-Protein Signaling 7 (*RGS7*)], regulation of apoptosis [Thymocyte Nuclear Protein 1 (*THYN1/THY28*) and Modulator of apoptosis protein 1 (*MOAP1*)] (Toyota et al., 2012), and α-synuclein aggregation [Secretogranin V (7B2 Protein) (*SCG5*)] (Helwig et al., 2013). Moreover, the protein products of 7 of the 11 genes in the putamen DE list interact with Ubiquitin (UBC) [Biogrid (http://thebiogrid.org/)]. GABARAPL1 and TSPYL1 proteins both interact with sirtuin 1 (SIRT1), a NAD-dependent deacetylase attributed with neuroprotecive activities in PD, AD, and HD (reviewed in Herskovits and Guarente, 2014), which is required for resveratrol-mediated induction of autophagy (Wu et al., 2011).

Notably, 6 of the 11 genes decreased with PD in neurons within the putamen are also decreased in PD cortical neurons (*BEX1, ATP6V1E1, RGS7, MDH1, THYN1, MOAP1*). There were also expression changes shared between the putamen and substantia nigra, including the decreased expression of the autophagy-related gene *GABARAPL1* and lysosome H+ transporting ATPase subunits (*ATP6V1E1* and *ATP6V1A* in putamen and substantia nigra respectively). There were also trends toward DE of *MDH1* (*p* = 0.06), *MOAP1* (*p* = 0.113), and *BEX1* (*p* = 0.232) in the substantia nigra. It is also noteworthy that SNARE and NSF proteins (found decreased by PSEA and in LMD studies of PD substantia nigra neurons, see above) have been recently implicated in autophagy (Moreau et al., 2013) in addition to their better characterized function of being essential for vesicular fusion at the plasma membrane (Sudhof, 1995).

## Summary and conclusions

Our study has further demonstrated the applicability and utility of PSEA to refine analyses of interesting human disease datasets. These further demonstrate the technical soundness of the method and show solutions for applying PSEA in cases where modeling all resident cell populations simultaneously is unfeasible. Most importantly, we show how PSEA can be applied to generate and refine hypotheses regarding the etiopathology of human neurological disorders, thereby contributing to the larger efforts to find new therapies. Our PSEA analyses were able to bring cell-type-specific disease pathways into view in this study. Findings in PD neurons supported the growing evidence that autophagy is an important aspect of PD etiology and identify additional potential contributors to autophagic and mitophagic dysfunction. Together with the identification of NSF as a candidate PD susceptibility gene our data suggest NSF as a strong candidate for further analysis. In HD neurons, both expected and novel facets of endosomal and plasma membrane signaling processes showed dysregulation. Moreover, the fact *DCLK1* was detected as DE in both HD and PD neurons indicates that its potential involvement in neurodegenerative processes should be carefully considered.

It will be interesting in subsequent work to apply PSEA to other diseases and tissues. Moreover, we look forward to applying it to other data types, such as proteomic or metabolomic data, in which complementary insights into disease-related processes can be detected.

### Conflict of interest statement

The authors declare that the research was conducted in the absence of any commercial or financial relationships that could be construed as a potential conflict of interest.
